# Toward a standard in structural genome annotation for prokaryotes

**DOI:** 10.1186/s40793-015-0034-9

**Published:** 2015-07-25

**Authors:** H. James Tripp, Granger Sutton, Owen White, Jennifer Wortman, Amrita Pati, Natalia Mikhailova, Galina Ovchinnikova, Samuel H. Payne, Nikos C. Kyrpides, Natalia Ivanova

**Affiliations:** DOE Joint Genome Institute, Walnut Creek, California USA; J. Craig Venter Institute, Rockville, MD USA; Institute for Genome Sciences, University of Maryland School of Medicine, Baltimore, MD USA; Broad Institute, Cambridge, MA USA; Pacific Northwest National Laboratory, Richland, WA USA

## Abstract

**Background:**

In an effort to identify the best practice for finding genes in prokaryotic genomes and propose it as a standard for automated annotation pipelines, 1,004,576 peptides were collected from various publicly available resources, and were used as a basis to evaluate various gene-calling methods. The peptides came from 45 bacterial replicons with an average GC content from 31 % to 74 %, biased toward higher GC content genomes. Automated, manual, and semi-manual methods were used to tally errors in three widely used gene calling methods, as evidenced by peptides mapped outside the boundaries of called genes.

**Results:**

We found that the consensus set of identical genes predicted by the three methods constitutes only about 70 % of the genes predicted by each individual method (with start and stop required to coincide). Peptide data was useful for evaluating some of the differences between gene callers, but not reliable enough to make the results conclusive, due to limitations inherent in any proteogenomic study.

**Conclusions:**

A single, unambiguous, unanimous best practice did not emerge from this analysis, since the available proteomics data were not adequate to provide an objective measurement of differences in the accuracy between these methods. However, as a result of this study, software, reference data, and procedures have been better matched among participants, representing a step toward a much-needed standard. In the absence of sufficient amount of exprimental data to achieve a universal standard, our recommendation is that any of these methods can be used by the community, as long as a single method is employed across all datasets to be compared.

**Electronic supplementary material:**

The online version of this article (doi:10.1186/s40793-015-0034-9) contains supplementary material, which is available to authorized users.

## Background

As of July 13, 2013, more than a third of the 29,183 bacterial and archaeal genome sequencing projects listed in the Genomes On-line Database (GOLD) [1] are attributable to four major sequencing centers: DOE Joint Genome Institute (JGI, 4,250 projects), The Broad Institute (3,155 projects), J. Craig Venter Institute (JCVI, 1,976 projects), and Institute for Genome Sciences (IGS, 1,269 projects). Assuming an average of 3,000 gene predictions per genome for the 10,650 projects at these sequencing centers, an estimated 31,950,000 gene predictions will have been made by the completion of these projects. Given that each sequencing center has its own automated gene prediction pipeline, using software that has evolved separately over more than a decade, the question arises as to best current practices in structural genome annotation. In this context the phrase “structural gene annotation” refers only to finding the loci of protein-coding genes, not to annotating protein functions or predicting their 3D structure. Implementation of a single best practice would have the benefit of producing a single gene locus identifier for ease of cross-referencing in the scientific literature and for use by comparative genomics software [2]. A related motivation for this study was the need to consistently reannotate public genomes whose annotations are now more than a decade old.

Functional genomics data, such as RNA sequencing (RNA-Seq) and proteomics, provide a useful reference for evaluating and improving genome annotations [3–8]. A combination of the two is especially powerful, since RNA-seq data reveals transcript boundaries, whereas proteomics helps mapping translated sequences (coding sequences or CDSs). We found very few genomes where peptide data was available to confirm RNA-seq data, and since all available prokaryotic gene finders predict translated products rather than transcript boundaries, we explored whether proteomics alone could serve as a tool to identify a best practice for updating gene calls in outdated genome annotations.

A test set of genomes with varying GC% was identified and the gene calls for GeneMarkS [9], Glimmer3 [10], and Prodigal [11], which are the three most popular *ab initio* methods, were obtained from RefSeq’s public ftp site [12]. Peptides for most of the genomes were compiled from a PNNL website [8], with other data obtained from the PRIDE BioMart [13] and the publications of several independent research labs [6, 14, 15] (Additional file 1). We used the peptides to evaluate the accuracy of the GeneMarkS, Glimmer3, and Prodigal2.5 gene callers. In addition, we evaluated gene calling by the gene-finding post processor GenePRIMP [16] developed by JGI. Notably, the genome annotation versions of GeneMarkS, Glimmer3, Prodigal and GenePRIMP correspond to July 2013, when the work on this project was started. Based on the results of this work, reannotation of all public genomes integrated in IMG has begun at the JGI using the Prodigal based gene calling pipeline.

## Results

### Comparison of gene predictions

**Fig. 1:**
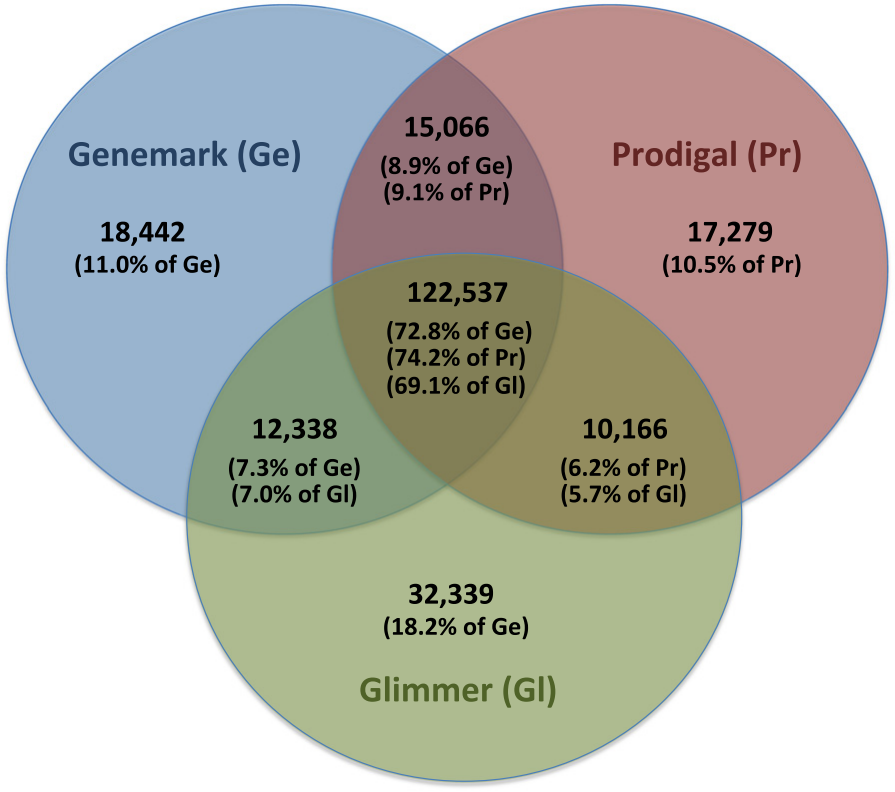
Overlaps between the sets of identical genes predicted by the three ab initio gene callers for 52 genomes. Gene predictions by two gene callers coincide only if both of their start and stop codons are predicted to be in the same positions on the same strand. The numerator for the percentages reported on the diagram is the number of relevant calls, which appears above the percentage. The denominator for the percentages is the total number of calls made by the gene caller, whose abbreviation appears after the percentage. Ge, GeneMark; Gl, Glimmer; Pr, Prodigal

The consensus set of identical genes (same strand, start, and stop) predicted by the three methods for 45 replicons (Additional file 1) constitutes only about 67-73 % of the genes predicted by each individual method, depending on which gene caller is chosen to provide the total number of calls on which the percentage is based (Fig. 1). The consensus set of genes, which vary only in their start codon, constitutes 83-96 % of the genes predicted by each individual method (data not shown). With respect to unique predictions, Glimmer3 made nearly twice as many as Prodigal and GeneMarkS did. With respect to agreement between pairs of gene callers, Prodigal and GeneMarkS agreed most often while Prodigal and Glimmer3 agreed the least.

### Peptide coverage of genes

Peptide coverage of predicted genes, which is to say the percentage of genes in the entire dataset that had at least one peptide mapping wholly inside of the gene, was on average approximately 40 % (data not shown). Total peptide support for gene calls, which is to say the total number of peptides that fell wholly inside of any gene prediction, was highest for Prodigal (1,000,574) and lowest for Glimmer3 (994,973) with GeneMarkS intermediate between the two (996,336).

### Comparison of detectible errors

**Fig. 2:**
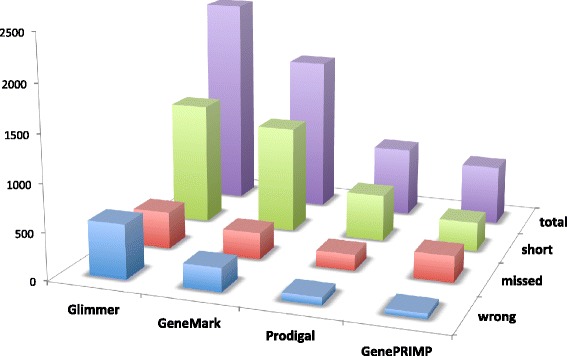
Overview of all gene calling errors by gene calling method. The number of gene calling errors found in the entire data set, by type, are plotted by gene calling method

Among *ab initio* gene callers, Glimmer3 scored the most errors in total and in each error category, Prodigal scored the fewest, and GeneMarkS scored intermediate between the two (Fig. 2). The GenePRIMP post-processor scored fewer total errors than any of the *ab initio* gene callers.

**Fig. 3:**
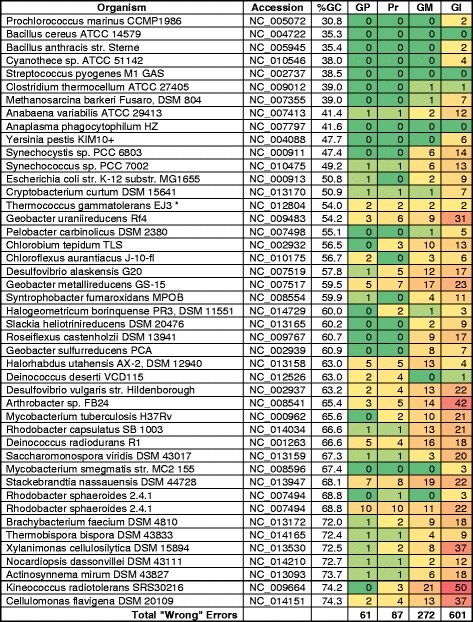
Total wrongly predicted (annotated) genes. GP, GenePRIMP; Pr, Prodigal; GM, GeneMarkS; Gl, Glimmer3

**Fig. 4:**
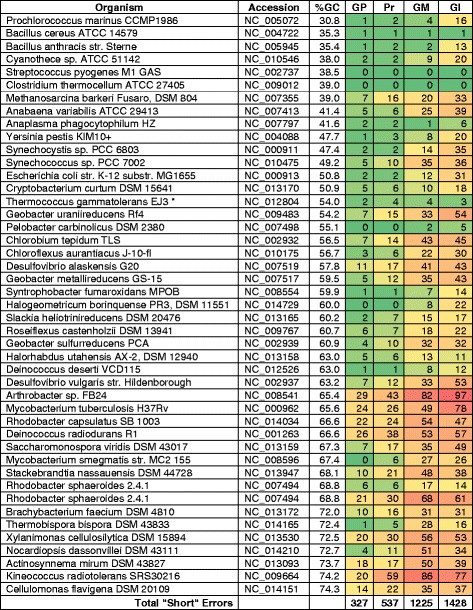
Genes with starts predicted downstream from detected starts (as indicated by proteomics). GP, GenePRIMP; Pr, Prodigal; GM, GeneMarkS; Gl, Glimmer3

**Fig. 5:**
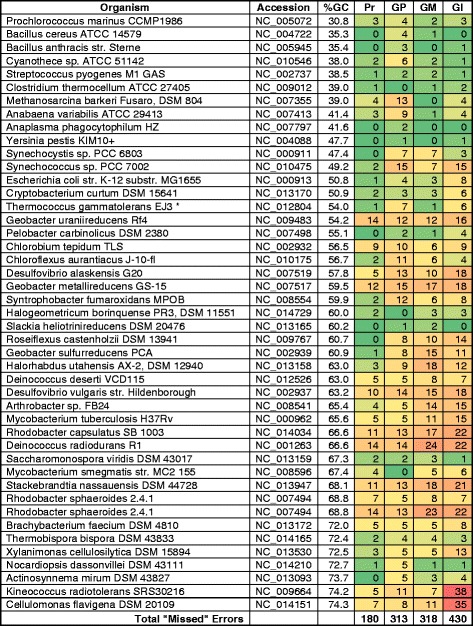
Genes missed by gene prediction (annotation) methods. Pr, Prodigal; GP, GenePRIMP; GM, GeneMarkS; Gl, Glimmer3

In an effort to gain a more detailed understanding of the summary results, heat maps of wrong, short, and missed gene calls plotted for each replicon (Figs. 3, 4, 5). Total wrong gene calls showed this pattern with respect to number of errors: Glimmer3 > GeneMarkS > Prodigal > GenePRIMP (Fig. 3). This pattern followed the pattern of the overall results. That is, GenePRIMP lowered the errors made by Prodigal and the two methods combine for the lowest errors. Glimmer3 had the highest number of errors for wrong gene calls. The results for total short gene calls follows the same pattern (Fig. 4). The results for total missed calls shows a different pattern: Glimmer3 > GeneMarkS > GenePRIMP > Prodigal (Fig. 5). The reason for this difference is the presence of genes with interrupted translation frames, which GenePRIMP identifies as pseudogenes and which is further addressed in the discussion.

## Discussion

A number of caveats must be kept in mind when attempting to estimate gene calling program performance from peptide data.

1. No proteomic experiment can guarantee expression of every gene in the genome.
2. Signal peptides, are often removed from proteins, making it impossible to guarantee peptide data pertaining to the true start site of translation.
3. Some peptide sequences, particularly highly hydrophobic ones, are not amenable to detection by mass spectrometry.
4. The mapping of peptide mass spectra to genome sequence may be erroneous and thus presents an opportunity for false positives.
5. It is impossible to detect “too long” errors in gene start calling using peptide data, since an error correcting peptide will never appear upstream of a predicted start that is already upstream of the true start. It is important to recognize that it is possible for a lower “short” gene error rate to be offset by a higher “long” gene error rate, resulting in a better overall rate of calling correct gene starts. So the “short” gene error rate in itself is not an unbiased measurement of a gene finder’s ability to choose correct gene start sites. However, considering that “short” gene errors prevent identification of functionally important conserved domains and motifs, and therefore can result in erroneous functional predictions, we report it here with this caveat in mind. In addition, we should point out that some genes have alternative translation initiation sites. This may have caused some spurious “short” errors, however all of the gene callers were under the same handicap in this regard.
6. It is impossible to detect false positive gene calls using peptide data, since peptides can only confirm gene calls; they cannot deny them.

In addition to these general caveats, it must also be reiterated that the genomes chosen for analysis are not a random, representative sample. Therefore, the results presented here must be considered an estimate of gene calling performance detectable with proteomics, not a definitive and absolute measurement of true gene calling performance.

At the same time, there is no definitive measurement of true gene calling performance against a randomly chosen, fully representative set of genomes. The biological knowledge to force expression of every protein in every genome does not exist, nor do high throughput biochemical methods for detecting every amino acid residue in every translation product in a cell, even if such knowledge of expression were available. The expression rate for this study, as measured by the peptide coverage reported above, averaged less than half (~40 %), but there is no reason to assume that this sample is systematically biased for or against any particular gene caller. Also, while it is true that peptides cannot detect false positive gene calls, statistical observations can give evidence of false positives: Glimmer3 made twice as many unique gene calls as Prodigal or GeneMarkS, but had the fewest number of confirming peptides. This does not prove that it makes more false positive predictions than the other gene callers; it simply offers some evidence that it might. High throughput proteomic data is the only option available for performing a wide survey of gene calling accuracy for thousands of genes in dozens of genomes. Use of high throughput proteomics is therefore an operational necessity if one wishes to perform a survey of gene-calling methods in preparation for a task such as reannotating all public genomes.

An important parameter affecting gene predictions made by *ab initio* gene callers is minimum gene length. Other things being equal, a shorter minimum gene length yields more candidate ORFs, which can result in a larger number of genes called. A biologically meaningful minimum gene length is 39 nucleotides (nt), which is the length of the PatS peptide, the shortest CDS yet detected [17]. However, such a short length generates so many spurious candidate ORFs that it is not recommended by designers of *ab initio* gene callers. The default minimum gene lengths recommended by program designers are: 90 nt for Prodigal, 81 nt for GeneMarkS, and 120 nt for Glimmer. These defaults were suggested by the developers of the corresponding tools to ensure their optimal performance. Selecting any other minimum gene length cutoff than 39 nt cannot be biologically justified but will undoubtedly result in poor performance; furthermore, it is likely to bias the analysis against one or another gene finder. For these reasons we chose to proceed with default cutoffs.

Turning to an analysis of the data regarding the three *ab initio* gene callers, it appears that Glimmer3’s “aggressive” algorithm for finding novel coding regions makes it prone to errors detectable with proteomics, while Prodigal’s design objective of eliminating false positives while retaining sensitivity makes it the least prone to such errors. The version of GeneMarkS tested, which now has been improved but not yet released, produced intermediate results. It is possible that the “aggressive” gene calling of Glimmer might be appropriate for a different set of genomes from novel single cell organisms. It may also be that the careful modeling of non-coding regions done by GeneMarkS (Mark Borodovsky, personal communication) may avoid more false positives than Prodigal in genomes with exceptionally low coding percentage and low GC content.

**Fig. 6:**
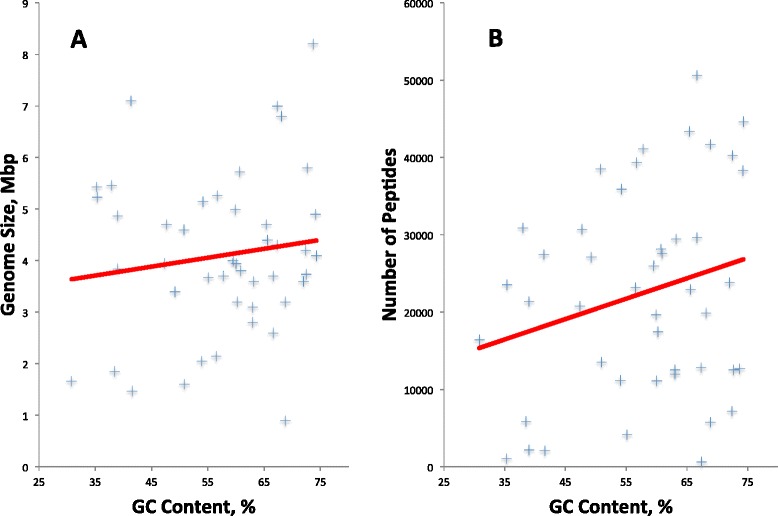
Bias toward increased genome length and number of peptides with increased GC content. **a**. The genome length in Mbp is plotted against GC content of replicons used. **b**. The number of peptides is plotted against GC content of replicons used

We explored the hypothesis that all gene callers might show poorer performance at high GC because they contain a higher frequency of alternate start codons and a lower frequency of stop codons. Our analysis uncovered two biases in our dataset that prevented rigorous exploration of this hypothesis: increased genome size with increasing GC content (Fig. 6a) and increased number of peptides with increasing GC content (Fig. 6b). Larger genomes are likely to have more total errors, and genomes with more peptides are more likely to have detectable errors. Future research might call for additional proteomics datasets generated with specific purpose of improving prokaryotic structural annotation by carefully selecting an unbiased set of genomes that will shed more light on the specific reasons for differences in performance of gene callers across genomes and for specific genomes.

**Fig. 7:**
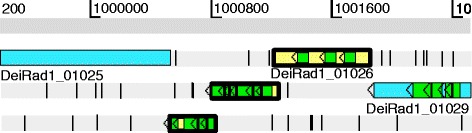
Artemis visualization of peptides refuting incorrect pseudogene call. The three boxes with thick black outlines and yellow backgrounds are CDS fragments of a single gene (DeiRad1_01026) disrupted by two frameshifts. The green boxes represent detected peptides

Although post processing by GenePRIMP generally improved upon Prodigal’s predictions, the number of missed genes was higher in GenePRIMP annotation due to the presence of pseudogenes. The issue of pseudogene calling does not arise with *ab initio* gene callers; they simply search for coding domains and make no attempt to analyze whether adjacent coding domains are part of a pseudogene. With regard to post processing by GenePRIMP, it must be noted that it does not automatically assume that all genes with frame disruptions are pseudogenes. Instead, it considers the number of frame disruptions (frameshifts and/or stop codons) and the length of the gene as compared to its homologs, and marks as pseudogenes only those with multiple frame disruptions and/or severe truncations. Furthermore, GenePRIMP retains the coordinates of all fragments of disrupted CDSs, even when they are annotated as pseudogenes. Frameshifted genes without a “pseudogene” tag that had confirming peptides were considered good calls despite their frameshifts, since the confirming peptides indicate that not calling the gene a pseudogene was a correct call even though frameshifts were present. On the other hand, GenePRIMP pseudogene calls with confirming peptides were scored as “missed,” since the confirming peptides indicate that the gene in question is a real gene, not a pseudogene, despite the fact that it may have multiple frameshifts due to sequencing and assembly errors (Fig. 7). This explains why missed gene errors for GenePRIMP are higher than Prodigal’s.

A similar situation occurs when sequencing error introduces an interrupted gene. GenePRIMP attempts to detect these instances and joins coding domains it deems to have likely been interrupted by the introduction of a spurious stop codon due to sequencing error. The partial coding domains are annotated as “exons,” even though they are not pieces of a gene interrupted by introns (as in eukaryotes), but rather pieces of a gene with spurious interruptions introduced by sequencing errors that result in frameshifts and internal stop codons. The missed gene data suggests that the GenePRIMP algorithm for detecting and annotating sequencing error is reliable and appropriate, albeit with a novel interpretation of “exon.” For similar reasons, NCBI has recently changed the guidelines for annotation of interrupted genes in RefSeq genomes (personal communication). Interrupted genes are annotated as partial coding regions if their translated protein products have significant similarity to full-length proteins in closely related genomes.

## Conclusions

Proteomics is a valuable aid to evaluating and improving gene-calling programs. When applied to 45 replicons of interest to the participants of this study, a combination of *ab initio* gene calling by Prodigal followed by GenePRIMP post processing had a lower estimated, operational error rate than GeneMarkS followed by Glimmer3. We have also compared these data against the RefSeq pipeline (version available in Spring 2013) and the results showed that the its overall performance was between that of GeneMark and Glimmer (data not shown). Nonetheless, due to inherent biologically-based limitations, we cannot conclude that proteomics alone should be used to define a best practice as the basis for a general standard in prokaryotic structural genome annotation; this must wait for better tools and expanded datasets that cover more taxonomic groups without biases in GC content, genome length, and gene expression levels. Some participants have already improved their pipelines, especially gene data models and reference databases, with the goal of one day achieving a much needed standard. Moving forward, a consensus approach, employing multiple gene callers and additional forms of expression verification such as RNA-seq, should be also explored as the possible basis for a standard in prokaryotic structural genome annotation.

## Methods

### Selection of genomes of interest

The genomes of interest had at least one of these characteristics:

1. The annotation was thought to be in need of updating.
2. The organism was well studied; preferably a type strain whose annotation had been heavily curated.
3. The genome added taxonomic diversity to the dataset.

Because of the genome selection criteria, the sample set is diverse and relevant to the participants, but it cannot be considered a representative random sample of genomes in nature or in public databases. The average GC content was 57.7 %, with a range of 30.8 % to 74.3 %. The average genome size was 4.3 Mbp with a range of 1.5 Mbp to 8.2 Mbp.

### Collection of data and loading to MySQL warehouse

A mySQL warehouse of proteogenomic information was created by acquiring, transforming, and loading publically available gene calls and proteomic data. The data sources for gene calls and peptides are shown in Additional file 1 and are available at http://portal.nersc.gov/dna/microbial/prokpubs/SIGS_proteogenomics/. GeneMarkS, Glimmer3, and Prodigal-2.5 in .gff format were downloaded from RefSeq public ftp site at the time of this study (Spring, 2013). These gene call coordinates were extracted from the .gff files and loaded into the warehouse. It must be noted that as a result of this study, development of a new version of GeneMarkS has started (GeneMarkS-2), however, predictions made by the new version have not been used in this study. The PNNL peptide data was also provided in .gff format, with mapping to its associated GenBank nucleotide sequence, allowing easy extraction and loading into MySQL. The non-PNNL peptide data was often not provided in .gff format, and sometimes did not have end coordinates. However, it always included individual peptide sequences, allowing each peptide to be mapped to its coordinates in the corresponding GenBank fasta file. Mapping was accomplished using a Perl script that searched for exact, unique matches in one of the six translation frames of the corresponding nucleotide sequence for the peptide. Short peptides that could not be mapped unambiguously were discarded. Unambigously mapped peptides were loaded into MySQL.

### Identification and analysis of peptides conflicting with gene calls

**Fig. 8:**
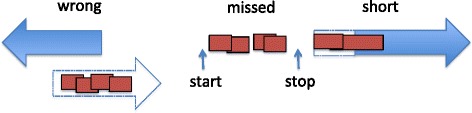
Schematic representation of scoring errors in gene calling. Right and left pointing arrows indicate genes called on positive and negative genome strand respectively. Boxes represent peptides detected by proteomics. Dashed contours show the extension of a gene or missed gene implied by peptide data

A SQL script identified peptides whose boundaries were partially or fully outside of a gene call. A Perl script produced a .gff file of conflicting peptides for each gene calling method. This .gff file of peptides and its corresponding nucleotide sequence were loaded into Artemis. A JGI Quality Assurance Analyst scored the gene associated with the conflicting peptide as wrong, missed, or short (Fig. 8). When conflicting peptides lay in the wrong reading frame relative to the gene call, the gene call was scored as “wrong.” When at least two conflicting peptides extended upstream of the predicted start site, the gene call was scored “short.” When conflicting peptides were in a region without any gene calls the implied gene was scored as “missed.” The false discovery rate (FDR) for the majority of the data was reported as 0.3 % [8]. In order to further reduce the rate of false positives, we required that each missed, wrong or short gene was detected by at least 2 non-redundant peptides, which would reduce the false positive rate to 0.09 %. All missed, wrong and short genes were additionally verified by BLASTp with an e-value of 1.0e-05 and by conserved motif and domain analysis.

## Additional file

Additional file 1:
**Replicons Used in This Study.** This is an Excel file listing the GenBank accession numbers of the 45 replicons used in this study, along with the number of peptides, the RefSeq source for gene calls, and the file name and publication reference for the peptide sources.
